# Supplementary material for: Transcriptomic Insights into Mechanisms of Early Seed Maturation in the Garden Pea (Pisum sativum L.)
Source: Cells. 2020 Mar 23;9(3):779. doi: 10.3390/cells9030779 (PMC7140803; doi:10.3390/cells9030779)

Module correlations (WTO algorithm)

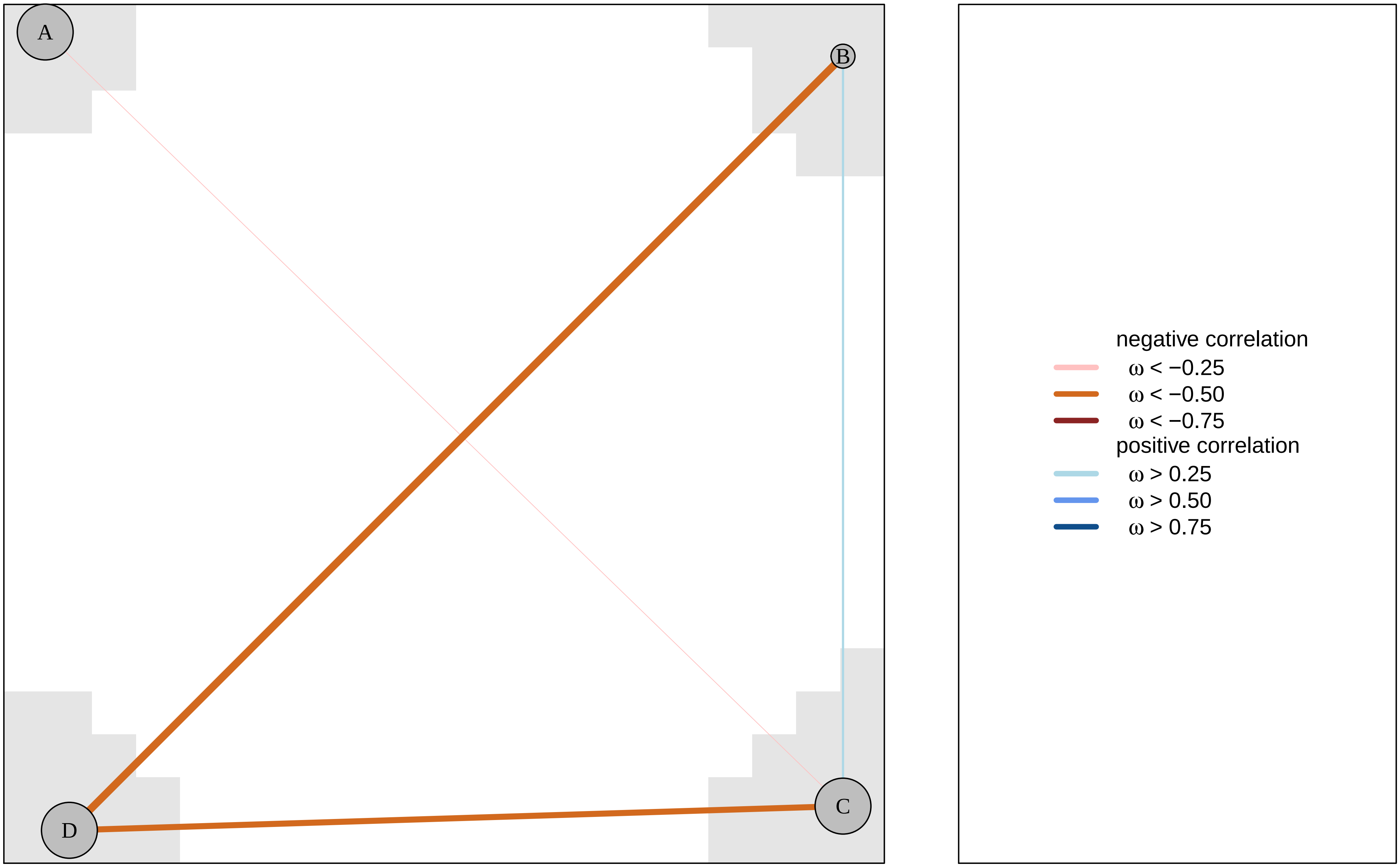

Module implications (basket algorithm)

all samples

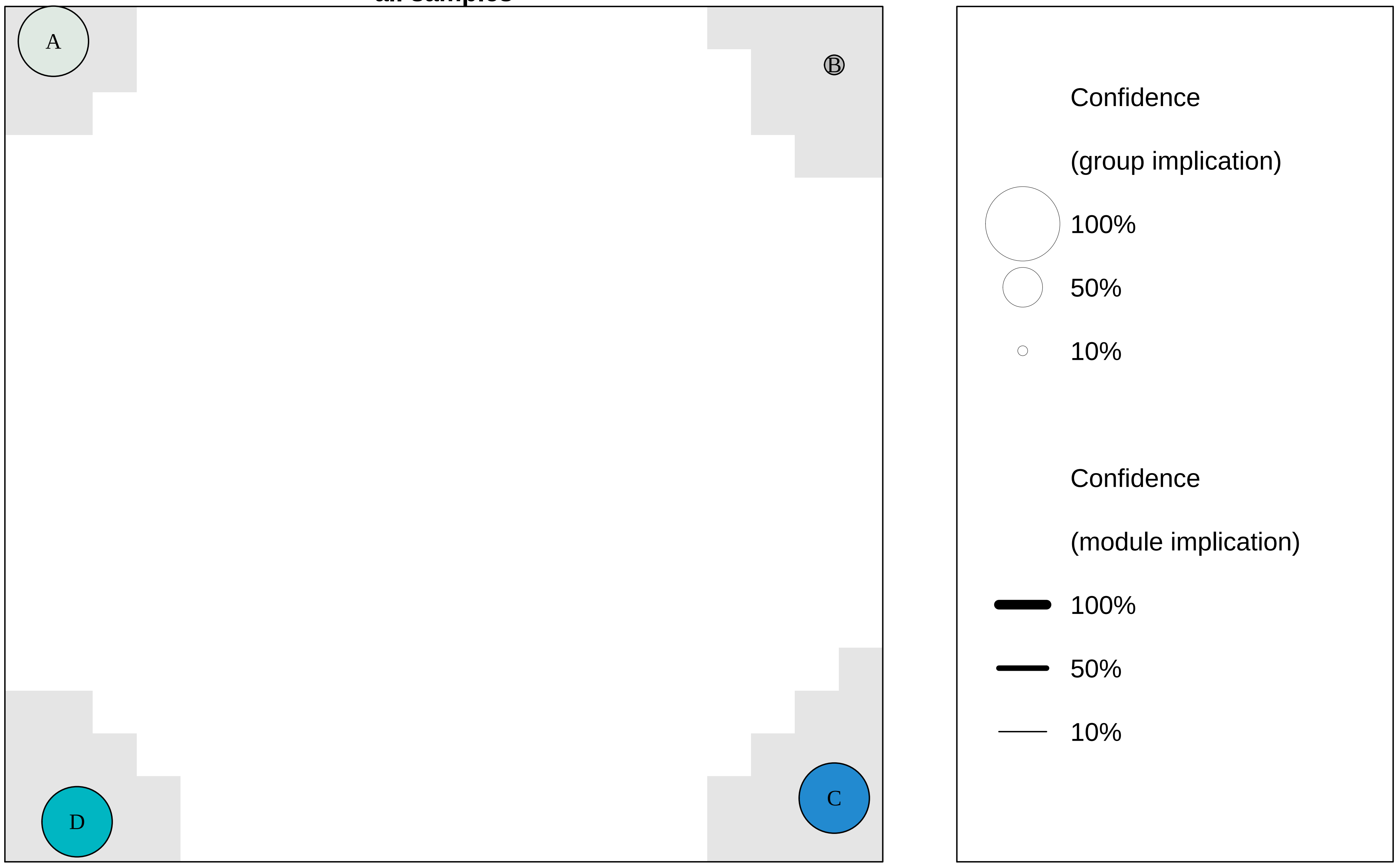

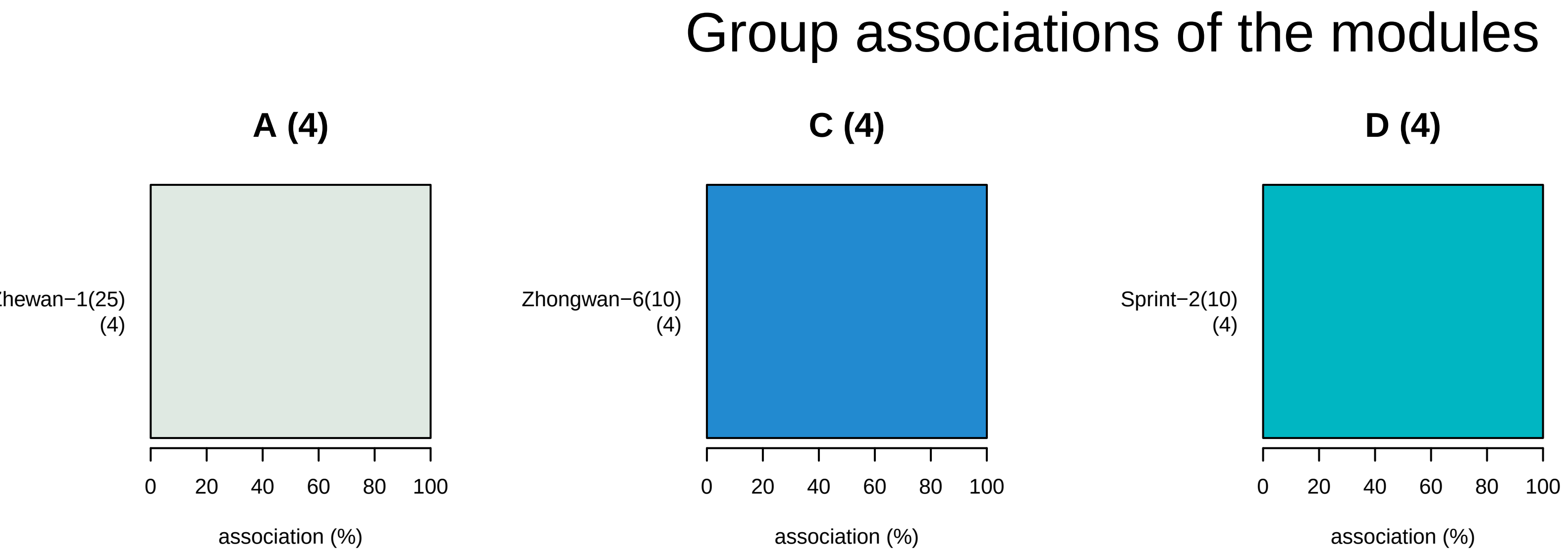
Group associations of the modules
A (4)
C (4)
D (4)
hewan−1(25)
(4)
Zhongwan−6(10)
(4)
Sprint−2(10)
(4)
0 20 40 60 80 100
association (%)
0 20 40 60 80 100
association (%)
0 20 40 60 80 100
association (%)

Module implications of the groups (basket algorithm)

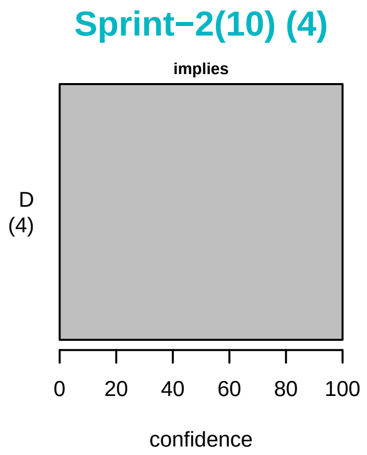

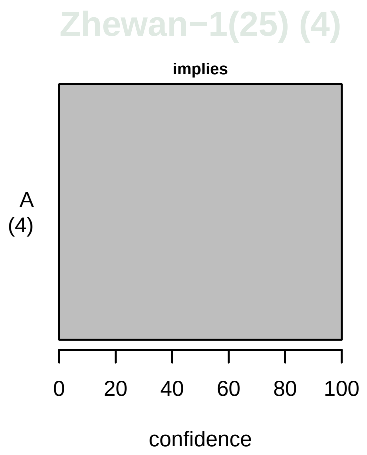

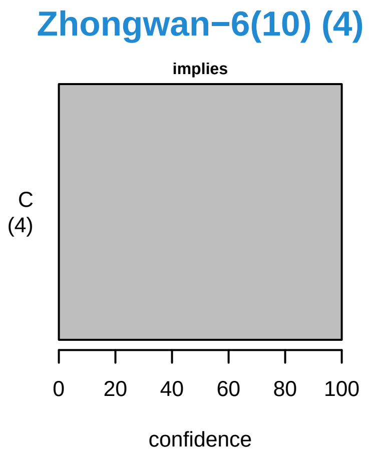

Group implications of the modules (basket algorithm)

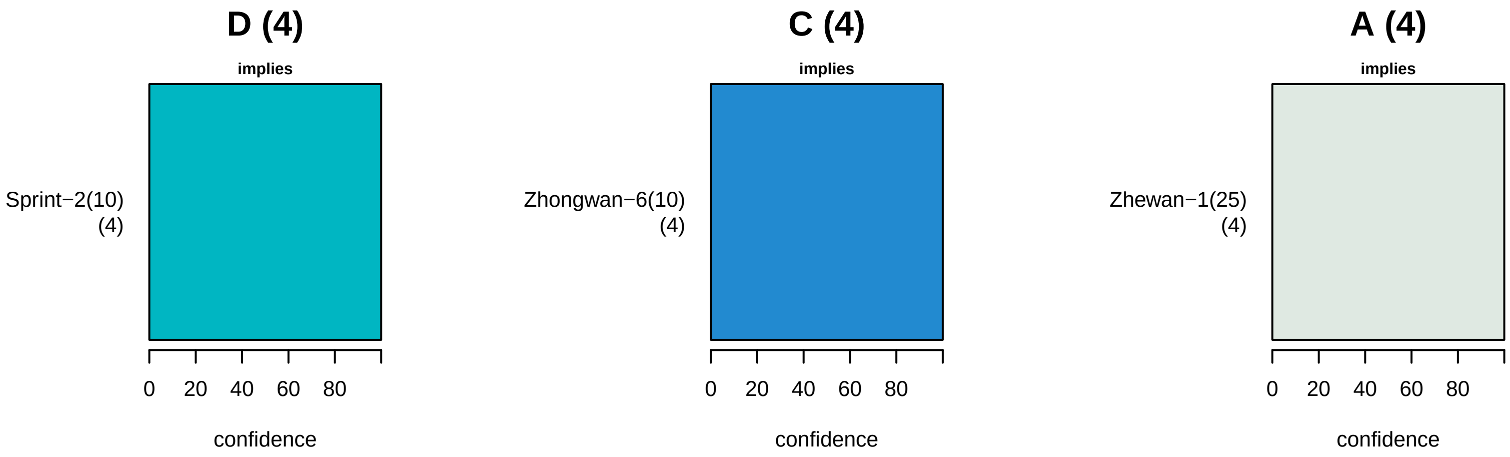

Supplement: Supplementary file 1 [file cells-09-00779-s001.zip › Supplementary materials/FileS3.zip!/FileS2/Hormones - Results/Summary Sheets - Modules/D-Cluster/Relations.pdf]
